# 2nd International Salzburg Conference on Neurorecovery (ISCN 2013) Salzburg/ Austria | November 28th - 29th, 2013

**Published:** 2014

**Authors:** M Brainin, D Muresanu, D Slavoaca

**Affiliations:** * Department of Clinical Neurosciences, Donau-Universität Krems, Krems, Austria; ** “RoNeuro” Institute for Neurological Research and Diagnostic, Cluj-Napoca, Romania; *** “Iuliu Hațieganu” University of Medicine and Pharmacy, Department of Clinical Neurosciences, Cluj-Napoca, Romania

**Keywords:** Salzburg Conference, neurorecovery, neurorehabilitation, stroke, dementia

## Abstract

The 2nd International Salzburg Conference on Neurorecovery was held on the 28th and 29th of November, 2013, in Salzburg, one of the most beautiful cities in Austria, which is well known for its rich cultural heritage, world-famous music and beautiful surrounding landscapes. The aim of the conference was to discuss the progress in the field of neurorecovery. The conference brought together internationally renowned scientists and clinicians, who described the clinical and therapeutic relevance of translational research and its applications in neurorehabilitation.

The 2nd International Salzburg Conference on Neurorecovery was held on the 28th and 29th of November, 2013, in Salzburg, one of the most beautiful cities in Austria, which is well known for its rich cultural heritage, world-famous music and beautiful surrounding landscapes. The aim of the conference was to discuss the progress in the field of neurorecovery. The conference brought together internationally renowned scientists and clinicians, who described the clinical and therapeutic relevance of translational research and its applications in neurorehabilitation. Their scientific reports discussed the basic science and clinical importance of neurorecovery, particularly in the fields of stroke and dementia, and clarified the relevant issues and challenges associated with ischemic and degenerative changes in the brain.

On the first day, Professor Reinhold Schmidt, currently the Vice Chairman of the Department of Neurology at Karl-Franzens University in Graz, Austria, opened the conference with his lecture. Professor Schmidt described two eras in the history of the Alzheimer's disease and vascular dementia as clinically recognized entities: the era of dementia discovery and the era in which the simple classification of dementia as either multi-infarct or post-stroke dementia has given way to criteria that recognize the heterogeneity of the vascular lesions leading to dementia. Professor Schmidt explained the reason why a new concept of dementia was necessary: the rise of a mixed pathology as the leading cause of dementia. The concepts of vascular cognitive impairment and vascular dementia have been thoroughly investigated in both clinical and research settings. Knowledge of how changes in blood vessels induce a change in cognitive capacity and can lead to dementia has increased considerably in recent years. According to Professor Schmidt, research is ongoing related to whether changes in the vascular functions of the brain can, to some extent, give rise to neurodegenerative diseases such as the Alzheimer’s disease. This possibility is reinforced by the increasing amount of data suggesting that the risk factors linked to cardiovascular diseases are similar to those linked to Alzheimer’s disease [**1**-**8**].

On November 29, eleven talks were scheduled in three sessions. The first session, chaired by Professor Michael Brainin and Professor Martin Dichgans, included three lectures.

Professor Michael Chopp, Scientific Director of the Neuroscience Research Institute, Department of Neurology, Henry Ford Hospital, and Department of Physics, Oakland University, discussed new experimental and translational evidence in brain recovery after stroke. Professor Chopp analyzed the mechanisms underlying the potentiation of post-stroke functional recovery using cell-based and pharmacological agents, which amplify endogenous neurogenesis in the subventricular zone and angiogenesis in the border zone of ischemic lesions in animals. Professor Chopp demonstrated that injured brains can be stimulated to promote angiogenesis and neurogenesis, which are coupled restorative processes that contribute to functional recovery from stroke, and that MRI indices of these neurorestorative events are highly correlative with neurologic function and may be used for real-time monitoring of recovery from stroke [**9**-**11**].

During the same session, Martin Dichgans, Professor of Neurology and Director of the Institute for Stroke and Dementia Research at the University of Munich, presented his observations on the perilesional potential for recovery from stroke and the contribution of small subcortical infarcts and refined the novel concept of white matter hyperintensity penumbra. Professor Dichgans showed that a substantial proportion of small subcortical infarcts exhibit an ischemic penumbra with variable patterns and noted that the clinical relevance of this finding remains insufficiently explored [**12**-**16**].

The session was closed by Michael Brainin, Professor of Clinical Neurology at Danube University in Krems, Austria. Professor Brainin's lecture provided theoretical and clinical updates on the concept of vascular cognitive impairment and on the detection and prevention of post-stroke cognitive impairment. He noted that despite a high prevalence of post-stroke cognitive impairment, therapeutic possibilities are still limited. Stroke and dementia share the same cluster of modifiable risk factors. Thus, lifestyle interventions and strict adherence to medication may not only decrease the risk of a recurrent stroke but also the risk of a post-stroke cognitive decline. Evidence for the role of risk factor modification in the prevention of cognitive decline after stroke is scarce and comes mainly from observational studies. Thus, there is a need for more randomized clinical trials (RCTs) targeting the prevention of post-stroke dementia using lifestyle interventions and a multiple risk factor approach [**17**-**25**].

Session two was chaired by Kennedy Lees and Matthias Endres and included three key talks. Kennedy Lees, Professor of Cerebrovascular Medicine at the University of Glasgow, discussed the following challenges in the rehabilitation research: trials with small sample sizes, the diversity of outcomes, incomplete information and perceived ethical dimensions. In the opinion of Professor Lees, registry trials are potential sources of bias, but they can also offer useful findings and are ideal for rehabilitation studies in many respects: low costs, institutional approvals, speed of enrollment, ethical dimension, international participation, participant diversity, blinding, the ease with which study protocols are followed and therapy is recorded, and well-defined timelines and endpoints. RCTs remain the gold standard when practical. Both RCT and registry approaches can be strengthened by a central adjudication (which mitigates national- or center-specific scoring bias and provides a statistical advantage from multiple scoring) and assumption-free ordinal analysis. Agreement is needed with regard to common measures and common outcomes with which to interpret rehabilitation trials [**6**,**7**,**26**-**28**].

Bo Norrving, Professor of Neurology at Lund University, Sweden, and Chairman of the Cerebrovascular Disease ICD-11 Working Group, explained the rationale for revising ICD-10 and what this means for stroke and stroke recovery as clinical entities. ICD-11 proposes a single block of “Cerebrovascular Diseases” within Diseases of the Nervous System to reduce misclassification and improve the clarity and clinical usefulness of the classification of these diseases. According to this system, stroke will be an entity of its own rather than being considered a “circulatory” or “cardiovascular” disorder. This is an important change that should be useful for practitioners, researchers, consumers and administrators as well as policy makers and governments. 

Professor Natan Bornstein, head of the Stroke Unit, Department of Neurology Tel Aviv Sourasky Medical Center, demonstrated the importance of early and intensive mobilization after stroke, which can maximize the gain from therapy, accelerate recovery and improve functional outcomes. The combination of behavioral and pharmacological adjuvant therapies such as neurotrophic factors (e.g., Cerebrolysin) may have an additive or synergistic effect in promoting brain recovery [**12**,**29**-**35**].

Session three included five lectures and was chaired by Wolf-Dieter Heiss and Bo Norrving. Matthias Endres, Professor of Neurology at the Charité Medical University in Berlin, discussed the translational principles and how to apply them in stroke research. In his opinion, the past two decades have witnessed an enormous expansion in our understanding of events that determine the fate of brain cells following vessel occlusion. Cell death following brain ischemia is mediated by a complex interplay of a number of pathophysiologically distinct mechanisms. Both blood vessels and parenchyma have been implicated in this interplay, and their complex interactions define the fate of compromised tissues and cells. The improvement of reperfusion, the inhibition of secondary damage and the induction of endogenous protection and repair deserve increased efforts to overcome the “translational roadblock” that appears to exist in the stroke field. Importantly, new strategies are necessary to widen the time window for therapeutic intervention and may lead to the regeneration of lost functions after stroke [**36**].

In the next talk, Professor Wolf-Dieter Heiss, Director of the Max Planck Institute for Neurological Research in Cologne, Germany, focused on recent imaging findings that address the issue of plasticity and recovery with respect to language and motor function in stroke patients. Functional tomographic neuroimaging techniques are increasingly used to investigate this issue. These techniques are useful for identifying structures in the bilateral networks involved in the recovery of neurologic deficits and may help in the development and evaluation of rehabilitative strategies. Repetitive transcranial magnetic stimulation (RTMS) might be useful for the reduction of contralesional activation and thus may support the recovery of neurologic deficit [**37**-**41**].

Christian Enzinger, head of the Research Unit for Neuronal Repair and Plasticity in the Department of Neurology & Division of Neuroradiology at the Medical University of Graz, Austria, discussed the potential contribution of quantitative and functional magnetic resonance imaging (MRI) in predicting recovery after stroke. Professor Enzinger explained why these techniques provide objective insights into the functional and structural changes associated with (motor) recovery after stroke. Regarding the predictive value of MRI, he emphasized that the re-establishment of physiological functional patterns and the preservation of motor pathway integrity appear to be more important than the lesion size in recovery outcomes and that there is an effect of re-establishing these functional patterns over and above clinical measures. In Professor Enzinger’s opinion, future studies should also explore the optimal timing and intensity of interventions and the mode of action of therapies [**42**].

Professor Ludwig Aigner from the Institute of Molecular Regenerative Medicine at Paracelsus Medical University in Salzburg, discussed the interesting topic of molecular pathways in experimental cognitive research. According to Professor Aigner, in the central nervous system, aging resulted in a precipitous decline in neurogenesis and neuroinflammation, with concomitant impairments in cognitive functions. Professor Aigner stated that such impairments could be ameliorated by modulating the systemic milieu/microenvironment. In his opinion, targeting neurogenesis and neuroinflammation to restore cognitive functions in the elderly is a valid approach [**43**-**45**].

The last talk of the session was given by Professor Dafin Fior Muresanu, Chairman of the Department of Clinical Neurosciences at the University of Medicine and Pharmacy, Cluj-Napoca, Romania, and President of the Romanian Society of Neurology and the Society for the Study of Neuroprotection and Neuroplasticity. Professor Muresanu discussed the advances in pharmacological support in brain protection and recovery [**2**,**34**,**46**-**50**].

He stated that there is a need to identify therapeutic methods that are able to limit brain and spinal cord damage or enhance motor function recovery through neuroprotective and neurorestorative treatments administered at time points after six months post-stroke. Professor Muresanu noted that from a pharmacological perspective, focusing on molecules that are capable of mimicking the function of endogenous molecules with multimodal and pleiotropic neuroprotective effects is the best approach for neurorecovery, especially when such treatments are combined with intensive physical training. Biological agents (e.g., neurotrophic factors and related molecules) with modulating and multimodal effects are preferred pharmacological agents for brain protection and recovery because they usually also have pleiotropic neuroprotective effects, which enables these compounds to pharmacologically bridge acute neuroprotective processes with long-term recovery processes.

The day was closed with a roundtable discussion in which Matthias Enders, Wolf-Dieter Heiss, Ludwig Aigner and Dafin Muresanu participated. The discussion focused on the usefulness of experimental evidence for rehabilitation. An introductory statement was given by Michael Chopp, and the discussion was led by Natan Bornstein.

**Conflict of Interest**

The authors declare no conflict of interest.
